# Association of waist circumference with haemoglobin A1c and its optimal cutoff for identifying prediabetes and diabetes risk in the Chinese population

**DOI:** 10.1007/s11739-022-03072-z

**Published:** 2022-08-24

**Authors:** Juanying Zhen, Shuyun Liu, Guoru Zhao, Hao Peng, Nithushi Samaranayake, Aimin Xu, Chao Li, Jun Wu, Bernard Man Yung Cheung

**Affiliations:** 1 Department of Medicine, School of Clinical Medicine, The University of Hong Kong, Queen Mary Hospital, Pokfulam, Hong Kong SAR, China; 2 Department of Neurology, Peking University Shenzhen Hospital, Shenzhen, China; 3 Department of Neurology, Shenzhen Longhua District Central Hospital, Shenzhen, China; 4 CAS Key Laboratory of Human-Machine Intelligence-Synergy Systems, Research Center for Neural Engineering, Shenzhen Institute of Advanced Technology, Chinese Academy of Sciences, Shenzhen, China; 5 Department of Epidemiology, School of Public Health, Medical College of Soochow University, Suzhou, China; 6 Department of Pharmacy and Pharmaceutical Sciences, Faculty of Allied Health Sciences, University of Sri Jayewardenepura, Nugegoda, Sri Lanka; 7 State Key Laboratory of Pharmaceutical Biotechnology, The University of Hong Kong, Pokfulam, Hong Kong SAR, China; 8 Institute of Cardiovascular Science and Medicine, The University of Hong Kong, Hong Kong SAR, China

**Keywords:** Waist circumference, Central obesity, Haemoglobin A1c, Type 2 diabetes mellitus

## Abstract

Haemoglobin A1c (HbA1c) is a marker of glycaemic control in type 2 diabetes mellitus (T2DM). Increased waist circumference (WC) is known to be associated with T2DM. Therefore, we investigated the relationship of WC with HbA1c and explored its optimal cutoff for identifying prediabetes and diabetes risk. This study included 2339 participants between 18 and 84 years of age [mean (SD) age, 43.5 (11.9) years] with valid data on WC, HbA1c and related variables in the Shenzhen–Hong Kong United Network on Cardiovascular Disease study. Participants on anti-diabetic medications were excluded. Multiple linear regression was used to investigate the relationship between HbA1c and WC. Cutoff values of WC indicating an HbA1c level of 5.7% and 6.5% were also assessed using optimal binning. There was a significant linear relationship between WC and HbA1c in the overall population (*B* = 0.261, *P* < 0.001), men (*B* = 0.206, *P* < 0.001) and women (*B* = 0.311, *P* < 0.001). After adjustment for smoking, alcohol consumption, physical activity, hypertension, hypercholesterolaemia and age, the association remained significant in the overall population (*B* = 0.201, *P* < 0.001), men (*B* = 0.186, *P* < 0.001) and women (*B* = 0.182, *P* < 0.001). The optimal cutoff values of WC indicating an HbA1c level of 5.7% and 6.5% was 83 cm (entropy = 0.943) and 85 cm (entropy = 0.365) in men, and 78 cm (entropy = 0.922) and 86 cm (entropy = 0.256) in women. The linear relationship between WC and HbA1c in this study suggests that addressing central obesity issue is beneficial to people with T2DM or at risk of T2DM. WC cutoff values of 85 cm for men and 86 cm for women are appropriate for recommendation to undergo diabetes screening.

**Supplementary Information:**

The online version contains supplementary material available at 10.1007/s11739-022-03072-z.

## Introduction

Obesity is associated with increased risk of multiple diseases such as type 2 diabetes mellitus (T2DM), hypertension and cardiovascular disease, resulting in premature death. According to the latest national survey, more than half of Chinese adults are either overweight or obese, which have become a severe public health concern [1]. It suggested that overweight and obesity would increase the risk of major non-communicable diseases and premature mortality in the general population [1].

Haemoglobin A1c (HbA1c) can reflect average blood glucose level over the past 4 months because the average lifespan of erythrocytes is approximately 120 days. As HbA1c represents a weighted mean of glucose levels, it is used as an indicator of glycaemic control in patients with diabetes.

Waist circumference (WC) is a simple measure of central obesity, which is the cornerstone of the metabolic syndrome and an important risk factor for diabetes [2, 3]. Although glucose monitoring tools, like glucose monitoring meters and blood tests, are minimally invasive, WC is a more timesaving and less expensive screening tool. Therefore, WC may serve as an additional measure beyond blood glucose measurement for identifying prediabetic and diabetic risk. Central obesity has been shown to be associated with fasting plasma glucose (FPG) in Mediterranean, African and US populations [4–6]. An African study suggested that WC could predict incident dysglycaemia and T2DM in Black African men and women [5]. WC was associated with FPG in a US study [6]. Whether waist circumference (WC) is associated with HbA1c in Chinese has not been much investigated. Therefore, we aim to study the relationship between waist circumference and HbA1c in a Chinese population.

## Materials and methods

### Database and study population

The Shenzhen–Hong Kong United Network on Cardiovascular Disease is a population-based survey that began in December 2020. A total of 2339 participants from different communities in Shenzhen enrolled in the first batch of the survey at the end of 2020. The study was reviewed and approved by the institutional review board of Peking University Shenzhen Hospital and the University of Hong Kong. The survey comprises three parts—interview, physical examination and laboratory tests. Data on demographic characteristics, lifestyle factors, dietary habits, medical history and medication usage were obtained using a standard questionnaire during interview. All interviewers, physical examiners and phlebotomists completed training sessions to understand the standard methods and protocol of this survey and master the usage of the survey tools before conducting the survey. All participants voluntarily participated in this research and gave informed consent.

In the present study, participants who had valid data were included for investigating the association of WC with HbA1c and FPG. In all of them, valid data on WC, HbA1c, FPG, smoking, alcohol consumption, physical activity, blood pressure (BP), and hypercholesterolemia were available. Subjects who were on anti-diabetic medications were excluded from the study.

### Measurements and definitions

WC was measured in the horizontal plane midway between the lowest rib and the iliac crest using inelastic tape at the end of normal expiration. Central obesity was defined according to the WC cut-offs for Asian adults (WC ≥ 90 cm for male, WC ≥ 80 cm for female). BMI was calculated as the weight in kilograms divided by the square of height in meters (kg/m^2^). Smoking was defined as smoking any tobacco product, either daily or occasionally or having smoking history. Participants were categorised as drinkers if they reported that they drank any type of alcohol beverage at least once a week or had a history of alcohol drinking. Alcohol beverages included beer, liquor, wine and other alcohol beverages. Participants who had at least 30 min of moderate to high-intensive physical activity on 3 or more days a week were classified as physically active. Blood pressure was measured up to three times in the right arm using a mercury sphygmomanometer after at least 15 min of rest. Hypertension was defined as a previous diagnosis of hypertension by a doctor or another health professional, average blood pressure ≥ 140/90 mmHg, or taking medication for hypertension. Definition of hypercholesterolaemia was previous diagnosis of a doctor or other health professional, total serum cholesterol ≥ 200 mg/dL, or taking medication for hypercholesterolaemia. Blood samples were obtained to measure levels of FPG and HbA1c after fasting for 8 hours. Diabetes was defined by previous diagnosis from a doctor or other health professional, FPG ≥ 7.0 mmol/L, HbA1c ≥ 6.5% or taking medication for diabetes.

### Statistical analysis

Statistical analysis was carried out using SPSS Version 27 (IBM Corporation, Armonk, New York). Chi-squared test was used to analyse the baseline characteristics of participants. The FPG or HbA1c in different WC groups in different age groups (18–39 years, 40–59 years and 60–84 years) [7] in men and women were analysed using *t* test. Linear regression was used to evaluate the relationship of WC with FPG and HbA1c in men and women. Confounding factors such as age, hypercholesterolemia, hypertension, alcohol consumption, smoke and physical activity were included in the regression models. Further, we used optimal binning to find the cutoff values of WC indicating HbA1c of 5.7% and 6.5%. P ≤ 0.05 was considered significant .

## Results

A total of 2202 participants (951 men and 1251 women) aged 18–84 years were included in the analysis. In the sensitivity analysis, 152 participants with diabetes were excluded (Supplementary figure 1). In the overall population, 42% of participants had central obesity. The mean WC was 82.1 ± 10.2 cm.

Table 1 shows the general characteristics of the participants with respect to HbA1c. The mean WC of participants with HbA1c ≥ 6.5% was 89.9 ± 9.0 cm while the mean WC of participants with HbA1c < 6.5% was 81.7 ± 9.9 cm (*P* < 0.001). Participants with HbA1c ≥ 6.5% were older and had higher BMI (*P* < 0.001). Furthermore, participants with HbA1c ≥ 6.5% were more likely to be men (*P* = 0.018), had hypercholesterolaemia and hypertension (*P* < 0.001). However, there were no significant associations of HbA1c with smoking, alcohol consumption and physical activity.

**Table 1:** Characteristics of the study population with respect to HbA1c

	< 6.5%	≥ 6.5%	*P*
*n*	2068	134	
Age, years	42.9 ± 11.7	52.9 ± 11.4	< 0.001
Men	879 (42.5)	71 (47.0)	0.018
BMI, cm	24.2 ± 3.5	26.8 ± 4.6	< 0.001
Smoking	515 (24.9)	34 (25.4)	0.903
Alcohol consumption	569 (27.5)	33 (24.6)	0.467
Physical activity	1185 (57.3)	77 (57.5)	0.523
Hypercholesterolaemia	908 (36.4)	78 (58.9)	< 0.001
Hypertension	599 (29.0)	66 (49.3)	< 0.001
WC, cm	81.7 ± 9.9	89.9 ± 9.0	< 0.001

Data are presented as mean ± standard deviation or number (percent)

*HbA1c* haemoglobin A1c, *BMI* body mass index, *WC* waist circumference

Tables 2 and 3 show the difference in mean FPG or HbA1c in participants with and without central obesity. In men, mean HbA1c statistically differed between normal (WC < 90 cm) and central obese (WC ≥ 90 cm) subjects in the 18–39 age group (*P* < 0.001) and in the 40–59 age group (*P* = 0.017) but not in the 60–84 age group (*P* = 0.209). The differences were also found in women aged 18–39 years (*P* < 0.001), 40–59 years (*P* < 0.001) but not aged 60–84 years (*P* = 0.195). In addition, a difference in FPG level was found in male participants with and without central obesity in the 18–39 age group (*P* < 0.001) while a difference was found in women in the 18–39 age group (*P* = 0.025) and 40–59 age group (*P* < 0.001).

**Table 2:** Mean FPG, HbA1c in male participants with and without central obesity

Age	*n*	WC status	FPG mmol/L	*P*	HbA1c mmol/L	*P*
18–39	155	Central obese	5.35 ± 1.83	< 0.001	5.83 ± 1.16	< 0.001
	279	Normal	4.83 ± 0.50		5.44 ± 0.35	
40–59	172	Central obese	5.57 ± 2.04	0.092	5.99 ± 0.96	0.017
	259	Normal	5.26 ± 1.74		5.77 ± 0.93	
60–84	30	Central obese	5.46 ± 1.35	0.467	6.17 ± 1.01	0.735
	56	Normal	5.61 ± 2.09		6.00 ± 1.04	

Data are presented as mean ± standard deviation

*WC* waist circumference, *FPG* fasting plasma glucose, *HbA1c* haemoglobin A1c

**Table 3:** Mean FPG, HbA1c in female participants with and without central obesity

Age	*n*	WC status	FPG mmol/L	*P*	HbA1c mmol/L	*P*
18–39	129	Central obese	4.95 ± 0.79	0.025	5.55 ± 0.38	< 0.001
	388	Normal	4.77 ± 0.74		5.39 ± 0.44	
40–59	354	Central obese	5.25 ± 1.21	< 0.001	5.84 ± 0.80	< 0.001
	254	Normal	4.83 ± 0.49		5.56 ± 0.38	
60–84	90	Central obese	5.62 ± 1.19	0.165	6.16 ± 0.98	0.195
	36	Normal	5.30 ± 1.33		5.91 ± 0.63	

Data are presented as mean ± standard deviation

*WC* waist circumference, *FPG* fasting plasma glucose, *HbA1c* haemoglobin A1c

**Fig. 1:**
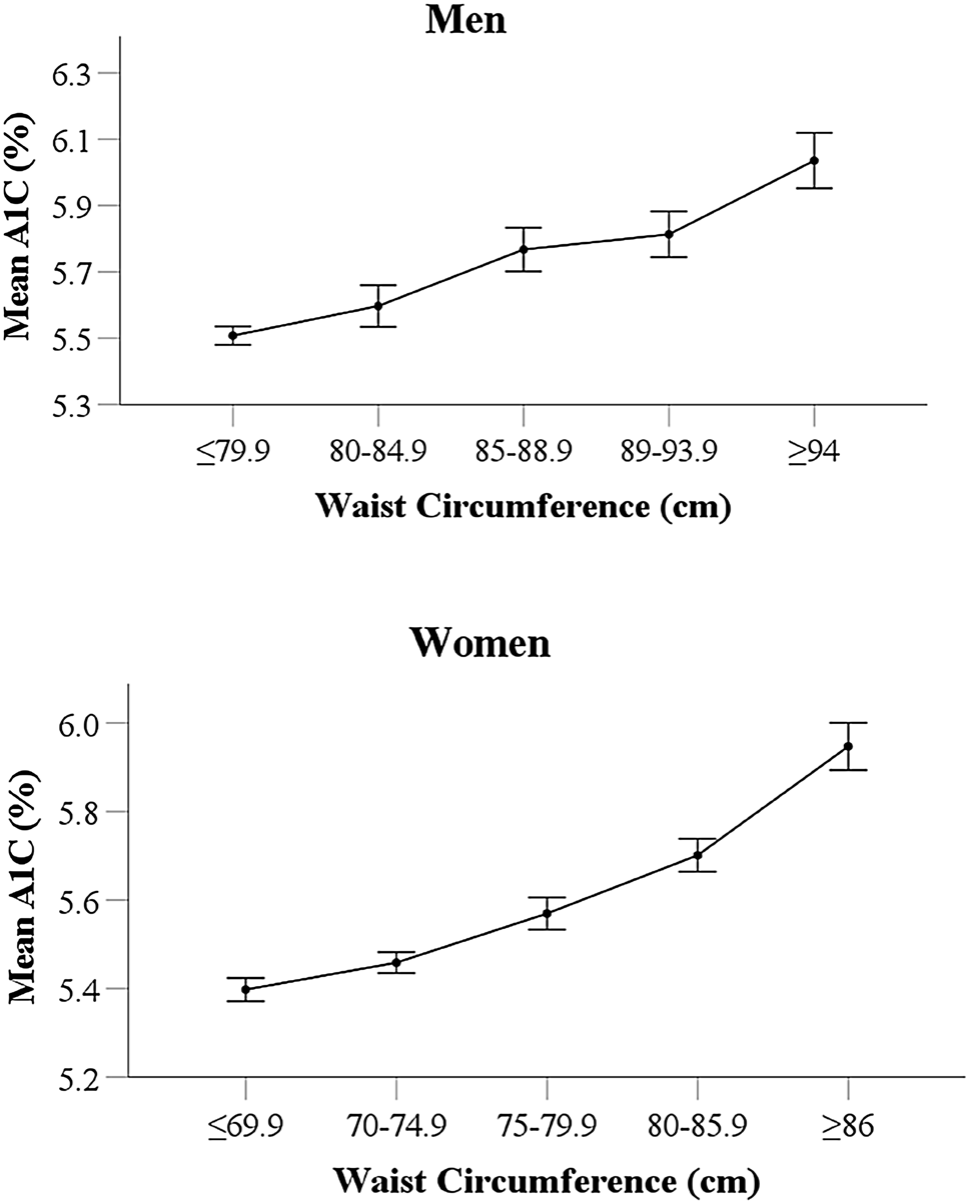
The linear relationship between waist circumference and HbA1c in men and women**.** HbA1c, Haemoglobin A1c. Error bars represent the standard errors

Figure 1 shows the linear relationship between WC and HbA1c in men and women. HbA1c was gradually increased with WC groups in men from 5.5% to 6.0% and women from 5.4% to 5.9%. Table 4 shows the linear regression analysis for the association between WC and HbA1c. In the linear regression model, WC was associated with HbA1c before adjustment in the overall population (*B* = 0.261, *P* < 0.001), in men (*B* = 0.206, *P* < 0.001) and women (*B* = 0.311, *P* < 0.001). After adjustment for smoking, alcohol consumption, physical activity, hypertension, hypercholesterolaemia and age, the association remained significant (overall: *B* = 0.201, *P* < 0.001; men: *B* = 0.186, *P* < 0.001; women: *B* = 0.182, *P* < 0.001).

**Table 4:** Association of WC with HbA1c in men and women

	HbA1c	
	B	*P*
Overall		
Unadjusted model	0.261	< 0.001
Model 1	0.250	< 0.001
Model 2	0.210	< 0.001
Model 3	0.201	< 0.001
Men		
Unadjusted model	0.206	< 0.001
Model 1	0.200	< 0.001
Model 2	0.186	< 0.001
Women		
Unadjusted model	0.311	< 0.001
Model 1	0.256	< 0.001
Model 2	0.182	< 0.001

Model 1: adjusted for smoking, alcohol consumption, physical activity, hypertension and hypercholesterolaemia

Model 2: further adjusted for age

Model 3: further adjusted for sex

Table 5 shows the optimal cutoff values of WC indicating HbA1c in men and women. In men, the optimal cutoff values of WC indicating an HbA1c of 5.7–6.5% was 83 cm (entropy: 0.943) and 85 cm (entropy: 0.365). In women, the optimal cutoff values of WC indicating an HbA1c of 5.7–6.5% was 78 cm (entropy: 0.922) and 86 cm (entropy: 0.256).

**Table 5:** The optimal cutoff values of WC indicating HbA1c in men and women

Gender	HbA1c	
	5.7%	6.5%
	WC (model entropy)	WC (model entropy)
Men	83 (0.943)	85 (0.365)
Women	78 (0.922)	86 (0.256)

*WC* waist circumference, *HbA1c* haemoglobin A1c

The linear regression analysis for the relationship between WC and FPG is shown in supplementary table 1. In the linear regression model, WC was associated with FPG before adjustment in the overall population (*B* = 0.221, *P* < 0.001), in men (*B* = 0.175, *P* < 0.001) and women (*B* = 0.259, *P* < 0.001). After adjustment for smoking, alcohol consumption, physical activity, hypertension, hypercholesterolaemia and age, the association remained significant (overall: *B* = 0.194, *P* < 0.001; men: *B* = 0.170, *P* < 0.001; women: *B* = 0.166, *P* < 0.001). WC was consistently associated with FPG after further adjusting for sex in the overall population (*B* = 0.182, *P* < 0.001).

In the sensitivity analysis (supplementary table 2), we excluded all diabetic patients to investigate the association of waist circumference with HbA1c in non-diabetic men and women. There was an association between WC and HbA1c before adjustment in the overall population (*B* = 0.230, *P* < 0.001), in men (*B* = 0.179, *P* < 0.001) and women (*B* = 0.270, *P* < 0.001). After adjusting for smoking, alcohol consumption, physical activity, hypertension, hypercholesterolaemia and age, the association was attenuated but still significant (overall: *B* = 0.162, *P* < 0.001; men: *B* = 0.141, *P* < 0.001; women: *B* = 0.158, *P* < 0.001). WC was consistently associated with FPG after adjusting for sex in overall population (*B* = 0.162, *P* < 0.001).

## Discussion

The main finding of this study is that there was a significant linear relationship of WC with HbA1c and FPG in Chinese aged 18–84 in the general population. The association was significant in both men and women after adjusting for smoking, alcohol consumption, physical activity, hypertension, hypercholesterolaemia and age.

Our study reveals a new and convenient way for people in general and diabetic patients to roughly know their HbA1c by measuring their WC. Frequent testing of FPG and HbA1c in non-diabetic persons causes inconvenience and discomfort. Measuring WC could be a supplementary method for regular use at home to prompt diet and lifestyle modification. In addition, our study is a community-based study. Shenzhen is a city of migrants and, therefore, the study population is representative of the general population in China, at least in terms of genetic background.

Previous studies in different Chinese populations have shown that WC, which is an index of central obesity, is significantly associated with T2DM. A longitudinal study in northern China involving 10,419 participants aged 20–80 years in 2008–2012 showed that WC and its changes were associated with the risk of T2DM. In Cox proportional hazard regression models, WC was found to be a risk factor predictive of T2DM, with an area under the receiver operating characteristic curve of 0.624, which was significantly greater than that for BMI. Moreover, the study found that every 2 kg weight gain or 3 cm WC increase conferred a 1.53-fold or 1.37-fold risk of developing diabetes, respectively [8]. In the Jackson Heart Study in the US that involved 2450 participants and a mean follow-up duration of 8 years, higher levels of HbA1c within the normal range were associated with incident diabetes (HR = 7.51, 95% CI 2.66–21.25) [9]. A large prospective study including 12,403 incident T2DM cases from eight European countries with 3.99 million person-years of follow-up reached the same conclusion, that WC was an independent risk factor for T2DM. The study further indicated that the association was stronger in women (HR = 31.8; 95% CI 25.2–40.2) than in men (HR = 22.0; 95% CI 14.3–33.8), which suggested that it is important to measure WC in women for risk stratification [10]. To date, most studies focused on the association between WC and diabetes but not on the association between WC and HbA1c. Our study highlights the significant association between WC and HbA1c in the general population.

Age differences in the association of WC with HbA1c were shown in our study. WC was significantly associated with HbA1c in the 18–39 age group in both men and women but the association was attenuated in the 40–59 age group and the 60–84 age group. Body adiposity changes with age, resulting in an increase in abdominal fat [11]. The changes of body composition with age may affect both WC and HbA1c. There are studies suggesting that obesity is a major driver in the development of T2DM in youth. A prospective study from the China Kadoorie Biobank that included 512,891 adult subjects aged 35–74 years demonstrated a significant association of central adiposity in early adulthood with incident T2DM after 9.2 years of follow-up [12]. A nationwide study of over one million Israelis aged 16–19 years showed that those with severe obesity had a higher risk of diabetes in the following decades [13]. Among severely obese boys and girls, the mean age of diabetes onset was 27.8 and 25.9 years, while the corresponding ages in the normal BMI reference group were 30.4 and 29.0 years [13]. These findings mean that central obesity increases diabetes risk as early as in adolescence rather than in old age. The measurement of WC allows early prediction of diabetes risk, and allows amelioration of that risk through lifestyle modification.

This study suggests that the optimal WC cutoff point indicating prediabetic risk for male and female are 83 cm and 78 cm whereas the optimal WC cutoff point indicating diabetic risk for male and female are 85–86 cm, respectively. The WC cutoff point indicating diabetic risk for female is higher than that for male. It may be relevant to recall that Japanese WC cut-offs identifying metabolic syndrome for men and women are 85–90 cm, which are equivalent to 100 cm^2^ of visceral fat. This discrepancy is due to the higher amount of subcutaneous fat in women for a given level of visceral adiposity [14]. T2DM is known to be more strongly associated with WC than BMI. As WC is an easy measurement for the general population, the optimal cutoff of WC for screening diabetes is of practical as well as reference value [15]. However, optimal WC cutoff points indicating prediabetic and diabetic risk are not much investigated. Moreover, the Chinese population is changing in terms of demographics, living standards and lifestyle, such that cutoff values based on previous data may no longer be applicable. Our study not only fills a void but also yields new optimal WC cutoff values based on up-to-date data on a Chinese population that is relatively young and affluent, and would, therefore, be predictive of future trends in China.

The limitation of our study is that the sensitivity and specificity for WC to identify diabetics is not high. However, WC is convenient for patients to measure at home and can be repeatedly measured over a long period of time. It can serve as an additional measure beyond blood glucose measurement at home between clinic visits.

## Conclusions

Our study suggests that there is a significant linear relationship between WC and HbA1c. Addressing central obesity issue is of great importance in people at diabetic risk or suffering from diabetes. WC cutoff values of 85 cm for men and 86 cm for women are appropriate for recommendation to undergo diabetes screening.

## Supplementary Information

Below is the link to the electronic supplementary material.

Supplementary file1 (DOCX 89 KB)

## Data Availability

The datasets analysed in the current study available from the corresponding author on reasonable request.
