# Supplementary material for: Association of waist circumference with haemoglobin A1c and its optimal cutoff for identifying prediabetes and diabetes risk in the Chinese population
Source: Intern Emerg Med. 2022 Aug 24;17(7):2039–44. doi: 10.1007/s11739-022-03072-z (PMC9522717; doi:10.1007/s11739-022-03072-z)
Supplement: Supplementary file 1 — Supplementary file1 (DOCX 89 KB) [file 11739_2022_3072_MOESM1_ESM.docx]

**Article title：Association of waist circumference with haemoglobin A1c and its** **optimal cutoff for identifying prediabetes and diabetes risk in the Chinese population**

**Journal name：**Internal and Emergency Medicine

**Author names：**Juanying Zhen, Shuyun Liu, Guoru Zhao, Hao Peng, Nithushi Samaranayake, Aimin Xu, Chao Li, Jun Wu, Bernard Man Yung Cheung

**Affiliation and e-mail address of the corresponding author:**

Prof Bernard Man Yung Cheung

Address: Department of Medicine, The University of Hong Kong, Queen Mary Hospital, Pokfulam Road, Hong Kong SAR

E-mail: mycheung@hku.hk

ORCID: 0000-0001-9106-7363

**Supplementary table 1**. Association of WC with FPG in men and women

|  | FPG |  |
| --- | --- | --- |
|  | B | P |
| Overall |  |  |
| Unadjusted model | 0.221 | <0.001 |
| Model 1 | 0.215 | <0.001 |
| Model 2 | 0.194 | <0.001 |
| Model 3 | 0.182 | <0.001 |
|  |  |  |
| Men |  |  |
| Unadjusted model | 0.175 | <0.001 |
| Model 1 | 0.179 | <0.001 |
| Model 2 | 0.170 | <0.001 |
|  |  |  |
| Women |  |  |
| Unadjusted model | 0.259 | <0.001 |
| Model 1 | 0.205 | <0.001 |
| Model 2 | 0.166 | <0.001 |

Model 1: adjusted for smoking, alcohol consumption, physical activity, hypertension and hypercholesterolaemia.

Model 2: further adjusted for age

Model 3: further adjusted for sex

**Supplementary Table 2**. Association of WC with HbA1c in non-diabetic men and women.

|  | HbA1c |  |
| --- | --- | --- |
|  | B | P |
| Overall |  |  |
| Unadjusted model | 0.230 | <0.001 |
| Model 1 | 0.206 | <0.001 |
| Model 2 | 0.162 | <0.001 |
| Model 3 | 0.162 | <0.001 |
|  |  |  |
| Men |  |  |
| Unadjusted model | 0.179 | <0.001 |
| Model 1 | 0.158 | <0.001 |
| Model 2 | 0.141 | <0.001 |
|  |  |  |
| Women |  |  |
| Unadjusted model | 0.270 | <0.001 |
| Model 1 | 0.227 | <0.001 |
| Model 2 | 0.158 | <0.001 |

Model 1: adjusted for smoking, alcohol consumption, physical activity, hypertension and hypercholesterolaemia.

Model 2: further adjusted for age

Model 3: further adjusted for sex

**
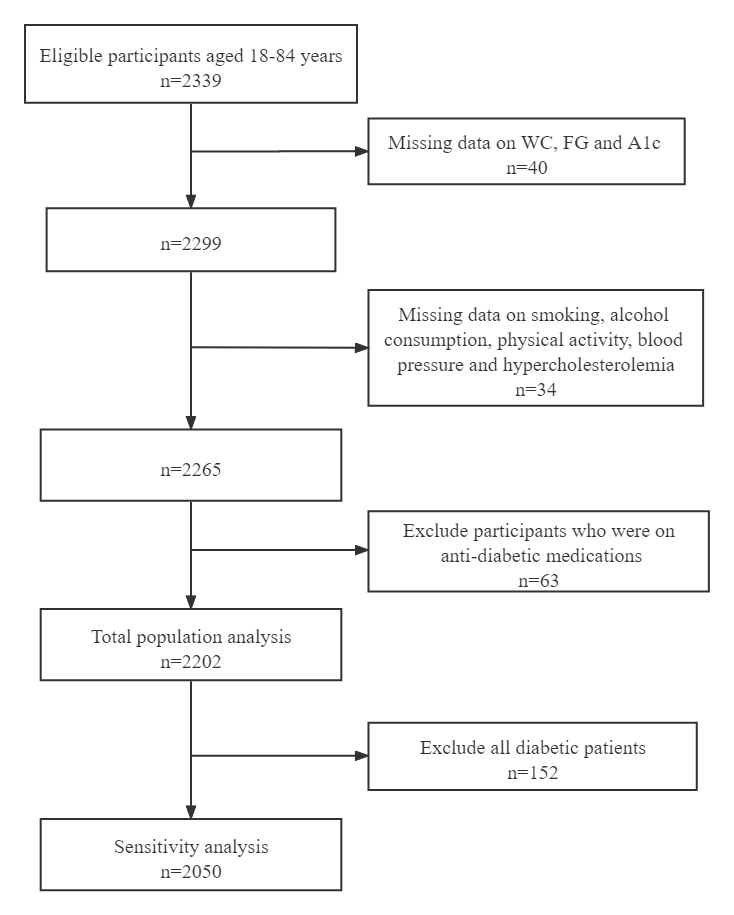
Supplementary figure 1.** Flowchart of study participants.
